# Application Effect of Computer-Assisted Local Anesthesia in Patient Operation

**DOI:** 10.1155/2021/8643867

**Published:** 2021-11-13

**Authors:** Yuchen Hao, Zheqi Zhang, Yan Meng

**Affiliations:** ^1^The Second Affiliated Hospital of Nanchang University, Nanchang, Jiangxi 330006, China; ^2^Shanxi Provincial People's Hospital, Taiyuan, Shanxi 030000, China; ^3^Zhangqiu People's Hospital, Jinan, Shandong 250200, China

## Abstract

In order to avoid the psychological harm caused by pain to patients, in this study, the application effect of computer-assisted local anesthesia in patient surgery was studied. In this method, 72 patients with hypertension, 35 males and 37 females, aged 53–83 years, with an average age of 70.8 ± 1.3 years, were selected for appointment tooth extraction in the department of stomatology from January to December 2014. All patients were booked for tooth extraction by ECG monitoring. Patients who were contraindicated for tooth extraction, had a history of mental illness, and had used antianxiety drugs and sedatives within 1 week before surgery were excluded. Patients were randomly divided into two groups according to their ID numbers: observation group, 36 cases, and control group. Painless oral local anesthesia injection instrument was used for local anesthesia injection. In the control group, 36 patients were injected with local anesthesia by traditional manual injection. The results showed that 86.11% of patients in the observation group had decreased anxiety scores after anesthesia, while only 13.88% of patients in the control group had decreased anxiety scores. Among patients with decreased anxiety scores, 80.65% in the observation group became nondental anxiety compared with 28.57% in the control group. Computer-assisted oral local anesthesia can effectively control dental anxiety and relieve the pain and discomfort of local anesthesia injection, and improve patient satisfaction, conducive to the smooth nursing work.

## 1. Introduction

With the improvement of people's living standards, patients have higher requirements for the comfort and safety of oral local anesthesia, especially for patients with dental anxiety [1]. Comfortable, safe, and painless oral local anesthesia is also the premise and guarantee for smooth oral treatment and also an effective means to relieve patients' fear, so as to avoid patients' dental anxiety and avoid oral treatment [2]. Dental anxiety refers to the patient's anxiety, tension, and fear of dental treatment. Tooth extraction is a common operation in alveolar surgery. The pain caused by local anesthesia injection is one of the important causes of dental anxiety in patients, which can lead to the excitation of the sympathetic nervous system, accelerated heart rate, and increased blood pressure. Especially for patients suffering from systemic diseases such as cardiovascular disease, the original diseases can be induced or aggravated, leading to serious complications [3]. Therefore, how to reduce the pain and fear of such patients, reduce the occurrence of dental anxiety, and reduce the risk of surgery have become problems to be solved at present [4]. Single tooth anesthesia is a computer-controlled local anesthesia injection instrument, which provides a new method for painless local anesthesia injection. Dental anxiety can lead to an increase in the secretion of endogenous adrenaline, thus improving the excitability of the sympathetic nerve, resulting in positive cardiac variability, manifested by accelerated heart rate and increased blood pressure [5]. It was observed that the blood pressure and heart rate of the experimental group were stable during the whole anesthesia process, while the blood pressure of the control group was significantly increased during the anesthesia injection. This suggests that STA computer-assisted oral local anesthesia can effectively reduce the risk of tooth extraction in such patients and reduce the occurrence of angina pectoris, myocardial infarction, arrhythmia, ventricular fibrillation, and other serious complications [6]. Pain is the main factor causing dental anxiety. In the clinical operation of stomatology department, it is accepted by more and more stomatological workers to avoid the psychological harm caused by pain. According to a large amount of clinical evidence, traditional local anesthesia injection can cause very obvious pain, thus aggravating patients' anxiety during and after treatment [7]. However, the anxiety level of hypertensive tooth extraction patients was the highest due to local injection and anesthetic effect. The usual way to reduce painful stimuli is to inject anesthetic drugs very slowly.

For this purpose, the computer-assisted oral local anesthesia instrument emerged as the times required. Due to its low injection speed, patients could hardly feel pain during the injection, thus achieving the purpose of reducing anxiety [8]. Hypertensive patients are a special group in oral therapy. Studies have shown that due to fear of pain in such patients, their preoperative anxiety level is significantly positively correlated with the increase of intraoperative blood pressure. Therefore, hypertensive patients may experience hypertensive crisis due to anxiety and pain during treatment [9]. For hypertensive patients with dental anxiety, pain relief and anxiety reduction are particularly important. In the treatment of tooth extraction assisted by computer-assisted oral local anesthesia, nurses need to cooperate with doctors to assist doctors to evaluate patients' dental anxiety. For patients with high MDAS scores, preoperative psychological counseling should be actively performed, and the whole process of treatment should be explained, especially the advantages of computer-assisted oral local anesthesia that can reduce pain and eliminate patients' fear of the instrument [10]. During the injection, assist the doctor to closely observe the patient's vital signs. Mn A. et al.'s study showed that 56% of elderly patients had different degrees of anxiety about dental treatment, most of which was caused by fear of pain. The level of anxiety before anesthesia was the highest in patients with cardiovascular disease due to local injection and anesthesia effect, and the level of anxiety before anesthesia was positively correlated with the increase of intraoperative blood pressure. Therefore, it is very important to use a painless local anesthesia injection technique for tooth extraction in cardiovascular patients with dental anxiety [11]. Ji H. L. et al. put forward the manual syringe with the needle tube. Since then, clinical oral local anesthesia has been following this traditional anesthesia injection method for more than a hundred years. Although the form and materials have been improved, the basic injection method is the same. The pain caused by the traditional manual injection mainly comes from the puncture pain caused by the puncture into the tissue and the pressure pain caused by the injection velocity. STA, a computer-controlled anesthesia system, was introduced to stomatology in 2007. The main principle is that the microprocessing chip in the host can automatically and accurately control the changes of injection pressure, flow rate, and other variables, so that the pressure of anesthesia drug injection is lower than the pain threshold of the body, so as to achieve the ideal effect [12]. Sreeja R. et al. believed that the slow and constant “slow flow rate” could significantly reduce the pain of patients with local anesthesia injection of palatal mucosa than “fast flow rate.” Second, STA uses a tubular package, which changes the traditional syringe style. The nonthreatening injection handle reduces the patient's expected anxiety. The introduction of anesthetics is controlled by the foot switch at the bottom of the machine, which changes the previous three-finger operation with one hand into pen-holding two-finger operation, and increased hand stability and flexibility. The rotating injection method avoids the deviation of injection position caused by the deflection force generated by the bevel angle of the needle and improves the accuracy of injection [13]. On the basis of the current research, this study proposed the application effect of computer-assisted local anesthesia in patient surgery. In this method, 72 patients with hypertension, 35 males and 37 females, aged 53–83 years, with an average of 70.8 ± 1.3 years, were selected for appointment tooth extraction in the department of stomatology from January to December 2014. All patients were booked for tooth extraction by ECG monitoring. Patients who were contraindicated for tooth extraction, had a history of mental illness, and had used antianxiety drugs and sedatives within 1 week before surgery were excluded. Patients were randomly divided into two groups according to their ID: observation group (*n* = 36) and control group. In the control group, 36 patients were injected with local anesthesia by traditional manual injection. The results showed that 86.11% of patients in the observation group had decreased anxiety scores after anesthesia, while only 13.88% of patients in the control group had decreased anxiety scores. Among patients with decreased anxiety scores, 80.65% in the observation group became nondental anxiety compared with 28.57% in the control group. In the monitoring of heart rate and blood pressure, this study found that the heart rate and blood pressure of the observation group did not change significantly before, during, and after injection, while the blood pressure of the control group increased significantly during local anesthesia injection compared with before and after injection. By reducing patients' injection pain and anxiety, changes in blood pressure and heart rate can be effectively controlled, so as to reduce the incidence of hypertensive crisis as much as possible.

## 2. Data and Methods

### 2.1. Clinical Data

A total of 72 hypertensive patients, 35 males and 37 females, aged from 53 to 83 years old, with an average of 70.8 ± 1.3 years old, were selected from the department of stomatology from January to December 2014. All patients were booked for tooth extraction by ECG monitoring. Patients who were contraindicated for tooth extraction, had a history of mental illness, and had used antianxiety drugs and sedatives within 1 week before surgery were excluded. Patients were randomly divided into two groups according to their ID: observation group (*n* = 36) and control group. In the control group, 36 patients were injected with local anesthesia by traditional manual injection.

### 2.2. Methods

#### 2.2.1. Operation Method

The observation group and the control group were treated with four-hand operation, and the anesthetic was articaine epinephrine injection. Computer-assisted oral local anesthesia was used in the observation group, and the local anesthesia apparatus was STA (single tooth anesthesia) produced by Milestone Company. The control group received traditional manual oral local anesthesia. During the whole tooth extraction process, patients in both groups were monitored continuously for blood pressure, heart rate, respiration, and oxygen saturation, and the doctors, nurses, and patients maintained verbal communication from the beginning to the end [14].

#### 2.2.2. Nursing Cooperation

Psychological nursing: patients are dental anxiety patients, and there is more need to understand their psychological state when seeing a doctor, targeted psychological nursing. Timely communication with patients and their families is particularly important, in the communication with patients to be warm, sincere, patient. All treatment and expenses should first obtain the understanding and informed consent of the patient and obtain the trust and cooperation of the patient. When introducing computer-assisted or traditional manual oral local anesthesia to patients and their families, try to combine physical objects, pictures, and texts to let them understand the operation method, process, and how to cooperate with medical staff. Relieve patients' anxiety and fear as far as possible, which is conducive to giving play to patients' subjective initiative and good treatment. During the whole operation, the patient was closely observed, always paying attention to the patient's own feelings. Before the implementation of each operation, inform patients in advance, so that they have the corresponding psychological preparation. Individual postoperative guidance should be given, and do a serious, meticulous follow-up. Nursing before local anesthesia: understand the patient's chief complaint, medical history, medication, anesthesia history, allergy history, and oral treatment history in detail and preliminarily formulate a thoughtful and reasonable nursing plan. Explain to the patient how to fill in the MDAS scale. Routine instruments and drugs for oral local anesthesia were prepared. The observation group needed to prepare STA painless oral local anesthesia instrument and ensure the normal operation of the equipment. Before, the observation group used STA for local anesthesia injection, and the working principle and advantages of STA must be explained to the patients to eliminate the tension of the patients. Lead the patient to sit in the dental chair, adjust the position and light, and connect the blood pressure and heart rate monitor. Local anesthesia nursing: the observation group and the control group were coordinated with the four-hand operation method, monitoring the blood pressure and heart rate of all patients and recording, asking patients to raise their hands if they have any discomfort, encouraging patients at any time to ensure the successful completion of local anesthesia and treatment, and paying attention to psychological nursing throughout the whole process. The control group received traditional manual oral local anesthesia. The observation group connected the STA oral painless local anesthesia instrument to the power supply. After the completion of the equipment self-test, pilin was loaded into the STA tube according to the routine, and the tube was connected to the STA device. After checking STA exhaust and cartridge capacity, insert the injection handle into the handle slot of the equipment for later use and adjust the injection speed of the equipment according to the doctor's injection requirements. After disinfecting the injection site, the doctor passes the injection handle to the nurse [15]. A cotton swab is placed slightly below the injection site prior to insertion to assist the physician with preanesthesia and/or suction of local anesthetic exudates to reduce discomfort caused by puncture and/or oral inflow. After the tip of the needle is inserted into the mucous membrane, the cotton swab can be withdrawn, while the patient's feelings and requirements are observed and questioned. Postoperative care of local anesthesia: after local anesthesia, the patients were asked about their feelings, using a 10-point visual analog scale (VAS), and the pain scores during local anesthesia were assessed by the patients themselves and recorded. MDAS scores and adverse reactions of local anesthesia were investigated, and patients in the observation group were asked whether they were willing to continue to choose computer-assisted oral local anesthesia for oral local anesthesia.

#### 2.2.3. Observations

Dental anxiety: dental anxiety scores were assessed by the MDAS scale before and after local anesthesia. MDAS consists of four questions, with options for each question including (a) relaxed, (b) a little uneasy, (c) and (d) be afraid or anxious, and (e) be so afraid or anxious that you sometimes sweat or feel unwell, count 1–5 points in order. MDAS ≥ 11 are considered as dental anxiety patients [16]. Blood pressure and heart rate: ECG monitor (UT4000 F) was used to measure patients' blood pressure and heart rate before, during, and after local anesthesia injection [17]. Pain degree: the visual analog scale (VAS) was used to evaluate the pain degree of patients. The VAS scale is a straight line with a length of 10 cm, with “0” and “10” at both ends representing “no pain” and “severe imagined pain,” respectively. Subjects mark the straight line according to their own pain situation, and the distance from the “no pain” end indicates the degree of pain. Adverse reactions of anesthesia: follow-up after anesthesia was conducted to investigate whether pain, swelling, hematoma, ulcer, tissue necrosis, and other adverse reactions occurred at the injection site [15].

#### 2.2.4. Statistical Methods

SPSS13.0 statistical software was used for data analysis. The measurement data with normal distribution and homogeneity of variance were expressed as mean ± standard deviation $\left(\bar{x}\pm s\right)$. The *t*-test was used for comparison between groups. Count data were expressed as frequency and percentage (%), and comparison between groups was performed by the *x*^2^ test. *P* < 0.05 was considered statistically significant.

## 3. Results and Analyses

**Figure 1:**
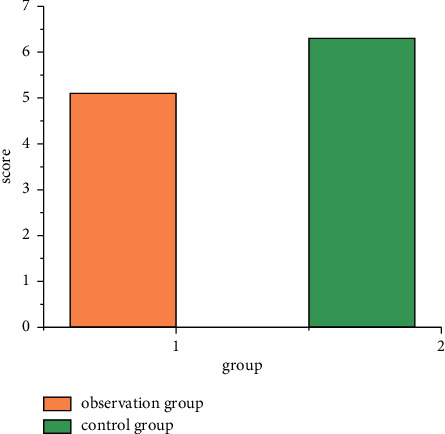
Comparison of pain degree of local anesthesia injection between the two groups.

Comparison of pain degree of local anesthesia injection between the two groups is shown in Figure 1: VAS score of the observation group was 0–6 points, with an average of 2.17 ± 1.859 points. The control group was 1–8 points, with an average of 3.67 ± 1.973 points [16]. VAS value in the observation group was lower than that in the control group, and the difference was statistically significant (*t* = 3.321, *P* = 0.001).

The comparison of dental anxiety between the two groups before and after local anesthesia is given in Table 1. After anesthesia, anxiety scores of 86.11% (31/36) patients in the observation group decreased, while only 13.88% (5/36) patients in the control group decreased, and the difference was statistically significant (*x*^2^  = 6.72, *P* < 0.01). In the observation group, 80.65% (25/31) patients' anxiety score decreased to nondental anxiety state (MADS < 11), while only 28.57% (4/14) in the control group, and the difference was statistically significant (*x*^2^ = 6.93, *P* < 0.01).

Comparison of blood pressure and heart rate between the two groups before, during, and after anesthesia is given in Tables 2 and 3. In the control group, blood pressure during local anesthesia injection increased compared with before and after anesthesia, with a statistical significance (*P* < 0.05), while heart rate accelerated, but the difference was not statistically significant (*P* > 0.05) [18]. There were no significant differences in blood pressure and heart rate between the observation group and the observation group before and after anesthesia (*P* > 0.05).

In the treatment of tooth extraction assisted by computer-assisted oral local anesthesia, nurses need to cooperate with doctors to assist doctors to evaluate patients' dental anxiety. For patients with high MDAS score, preoperative psychological counseling should be actively done, and the whole process of treatment should be explained. In particular, this study introduces the advantages of computer-assisted oral local anesthesia technology to reduce pain and eliminate patients' fear of the instrument. During the injection, assist the doctor to closely observe the patient's vital signs.

## 4. Conclusions

This study presents a study on the application effect of computer-assisted local anesthesia in patients undergoing surgery. This method selected 72 hypertensive patients, 35 males and 37 females, aged from 53 to 83 years old, with an average of 70.8 ± 1.3 years old, who underwent appointment tooth extraction in the department of stomatology from January to December 2014. All patients were booked for tooth extraction by ECG monitoring. Patients who were contraindicated for tooth extraction, had a history of mental illness, and had used antianxiety drugs and sedatives within 1 week before surgery were excluded. Patients were randomly divided into two groups according to their ID: observation group (*n* = 36) and control group. In the control group, 36 patients were injected with local anesthesia by traditional manual injection. The results show that 86.11% of patients in the observation group have decreased anxiety scores after anesthesia, while only 13.88% of patients in the control group have decreased anxiety scores. Among patients with decreased anxiety scores, 80.65% in the observation group became nondental anxiety compared with 28.57% in the control group. In the monitoring of heart rate and blood pressure, this study found that the heart rate and blood pressure of the observation group did not change significantly before, during, and after injection, while the blood pressure of the control group increased significantly during local anesthesia injection compared with before and after injection. By reducing patients' injection pain and anxiety, changes in blood pressure and heart rate can be effectively controlled, so as to reduce the incidence of hypertensive crisis as much as possible. In the future, other applications related to local anesthesia in computer-assisted surgery will continue.

## Figures and Tables

**Table 1:** Dental anxiety changes before and after anesthesia.

group	Example number	The score declined		Nondental anxiety state	
		Example number	Percentage	Example number	Percentage
Observation group	36	31	86.11	25/31	80.65
Control group	36	5	13.89	4/14	28.57

**Table 2:** Comparison of heart rate between the two groups before, during, and after anesthesia.

group	Example number	Heart rate		
		Before anesthesia	In anesthesia	After anesthesia
Observation group	36	71.89 ± 11.666	73.22 ± 11.086	72.78 ± 12.250
Control group	36	68.28 ± 11.860	71.51 ± 10.331	68.50 ± 10.025

**Table 3:** Comparison of blood pressure between the two groups before, during, and after anesthesia.

Group	Example number	Systolic pressure			Diastolic pressure		
		Before anesthesia	In anesthesia	After anesthesia	Before anesthesia	In anesthesia	After anesthesia
Observation group	36	134.39 ± 18.630	137.33 ± 18.346	132.29 ± 16.617	75.28 ± 10.932	78.06 ± 10.519	75.94 ± 10.378
Control group	36	141.39 ± 15.041	148.89 ± 17.019	139.5 ± 15.497	77.06 ± 11.476	82.39 ± 12.542	75.67 ± 10.754

## Data Availability

The data used to support the findings of this study are available from the corresponding author upon request.
